# Erosive pustular dermatosis-like eruption of the scalp secondary to amivantamab: A case series

**DOI:** 10.1016/j.jdcr.2023.11.027

**Published:** 2024-01-17

**Authors:** Mariya N. George, Riyad N.H. Seervai, Susan Y. Chon

**Affiliations:** a Department of Dermatology, University of Texas Health Science Center, McGovern Medical School at UTHealth Houston, Houston, Texas; b Department of Internal Medicine, Providence Portland Medical Center, Portland, Oregon; c Department of Dermatology, Oregon Health and Science University, Portland, Oregon; d Department of Dermatology, MD Anderson Cancer Center, Houston, Texas

**Keywords:** alopecia, amivantamab, EGFR inhibitors, erosive pustular dermatosis, immunotherapy, non–small cell lung cancer, oncodermatology

## Introduction

Amivantamab is a bispecific monoclonal antibody targeting epidermal growth factor receptor (EGFR) and mesenchymal-epithelial transition proto-oncogene approved in 2021 for treatment of locally advanced metastatic non–small cell lung cancer.^1^ Although cutaneous manifestations are common with EGFR inhibitors, erosive pustular dermatosis (EPD) of the scalp has not been reported with amivantamab to our knowledge. EPD of the scalp is a rare chronic eruption of crusted erosions or superficial ulcerations that can lead to scarring alopecia.^2^ EPD is typically associated with autoimmune disorders and known predisposing factors such as elderly age and photodamage.^3^ To our knowledge, we present the first reported cases of EPD-like eruption of the scalp associated with amivantamab treatment.

## Case 1

**Fig 1:**
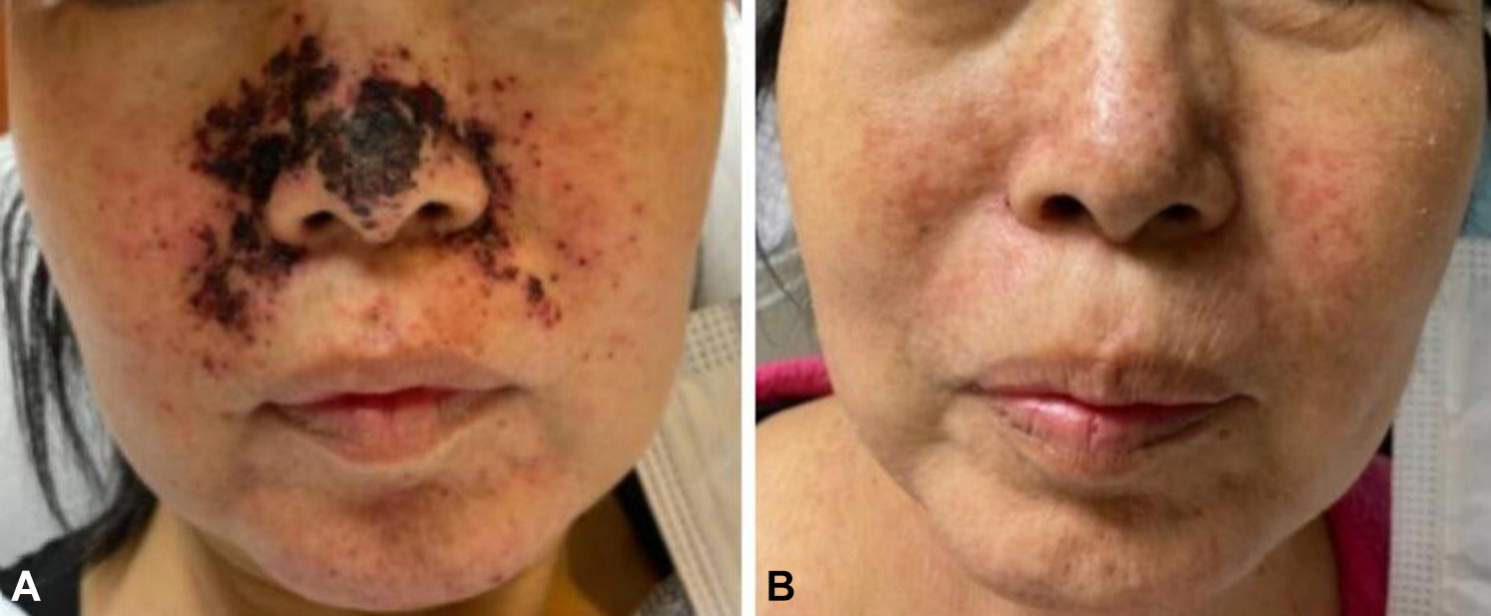
Case 1. **A,** Initial clinical presentation of acneiform rash. **B,** Improved acneiform rash 4 months into antibiotic treatment.

A 62-year-old woman with metastatic lung adenocarcinoma presented with a pruritic acneiform eruption on the face and scalp with paronychia and nailfold hypergranulation (Fig 1, *A*, *B*) 1 month after initiation of targeted therapy with amivantamab and osimertinib.

**Fig 2:**
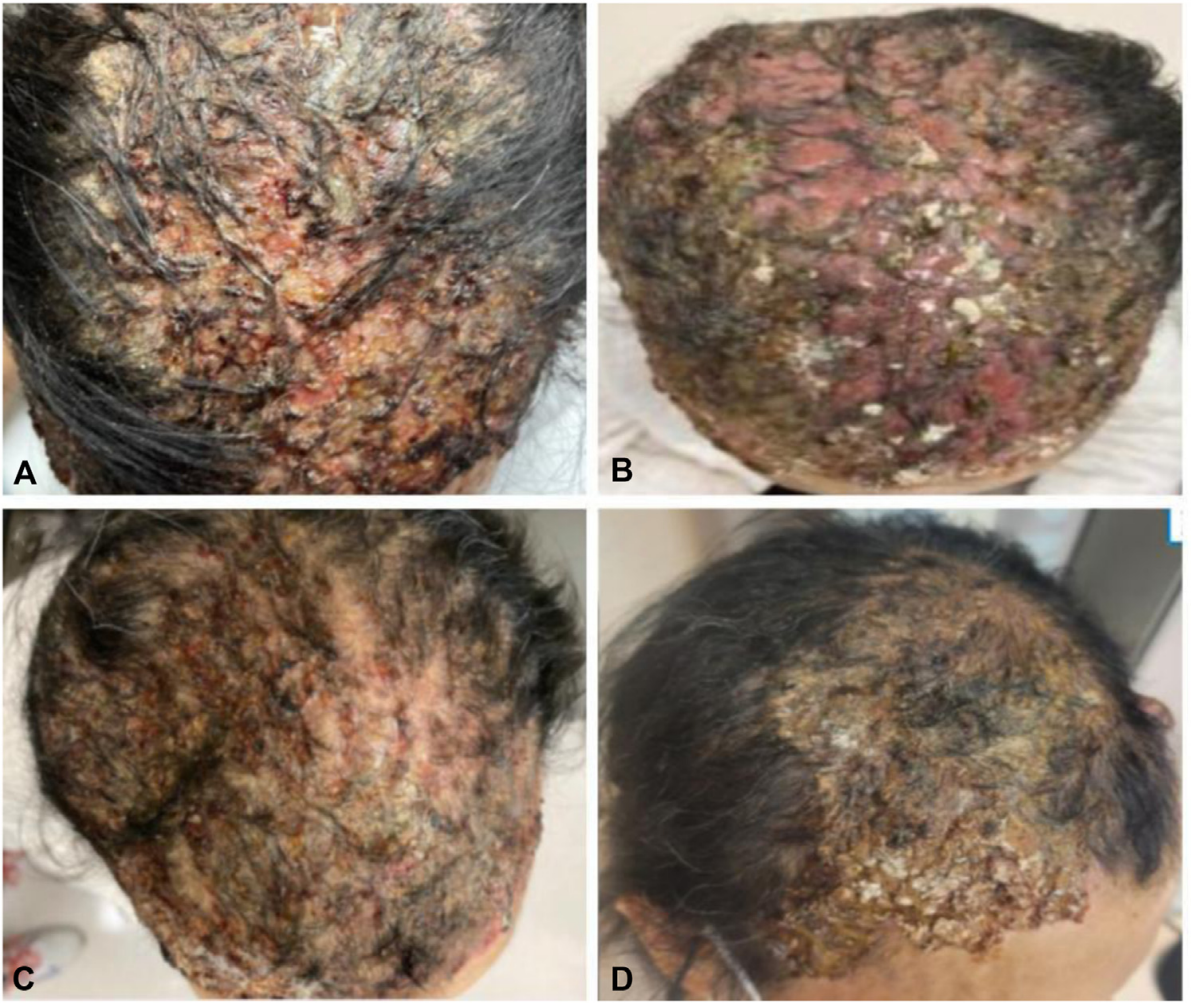
Case 1. **A,** Erosive pustular dermatosis-like eruption. **A**, Initial clinical presentation with crusted hemorrhagic erosions and pustules with alopecia on month 4 of treatment with amivantamab. **B,** Worsening symptoms with thick, yellow-brown crusts of erosions with alopecia on month 5. **C,** Improving symptoms after 3 week amivantamab holiday with decreased inflammation. **D,** Healed eruption with regrowth of hair 6 weeks after discontinuation of amivantamab.

Her symptoms improved with topical steroids and a combination of clindamycin lotion and doxycycline. However, 4 months into her treatment, she presented with worsening erosions and inflammation of the scalp with associated nonscarring alopecia (Fig 2).

**Fig 3:**
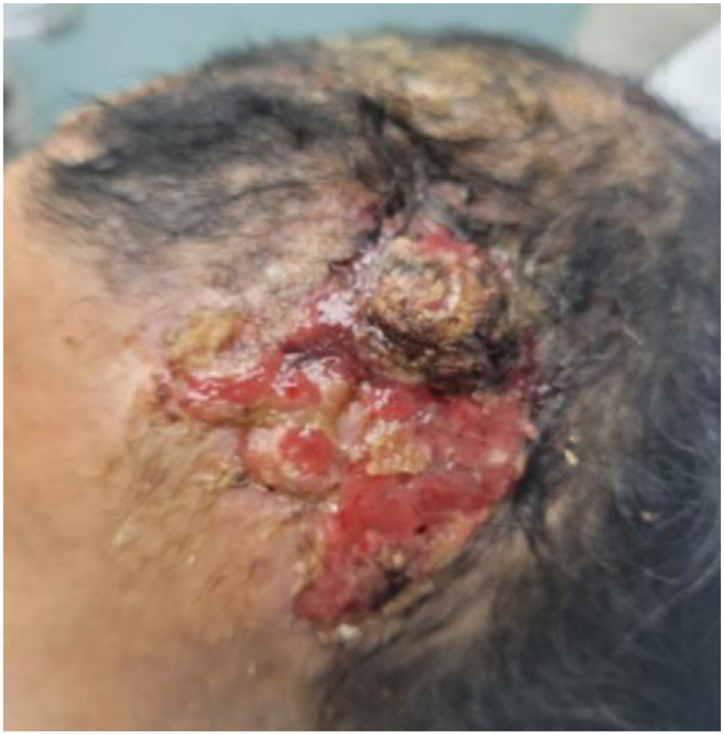
Case 1. Friable 2-cm nodule on the left side of the frontal aspect of the scalp present 1 month after discontinuation of amivantamab.

She had no known history of radiation or significant actinic damage to the scalp. Wound culture of the scalp showed normal-appearing skin flora. She was started on ketoconazole shampoo, fluocinolone oil, topical mupirocin, and multiple courses of doxycycline with no improvement in symptoms over the course of a month. At that point, she was started on prednisone and isotretinoin, and the amivantamab was held for 3 weeks. She experienced a marked improvement in symptoms, which again worsened after the reintroduction of amivantamab while continuing on the isotretinoin/prednisone regimen. The amivantamab was ultimately discontinued because of this severe skin toxicity and progression of her disease. She continued to have persistent toxicity from the amivantamab in the form of a 2-cm friable nodule that developed on the left side of the frontal aspect of the scalp a month following discontinuation (Fig 3), with biopsy showing fragments of granulation tissue with admixed naked hair shafts and an overlying crust/scale.

The patient was switched to carboplatin and pemetrexed for life-prolonging treatment, and her scalp completely healed with regrowth of her hair 3 months after the last dose of amivantamab.

## Case 2

**Fig 4:**
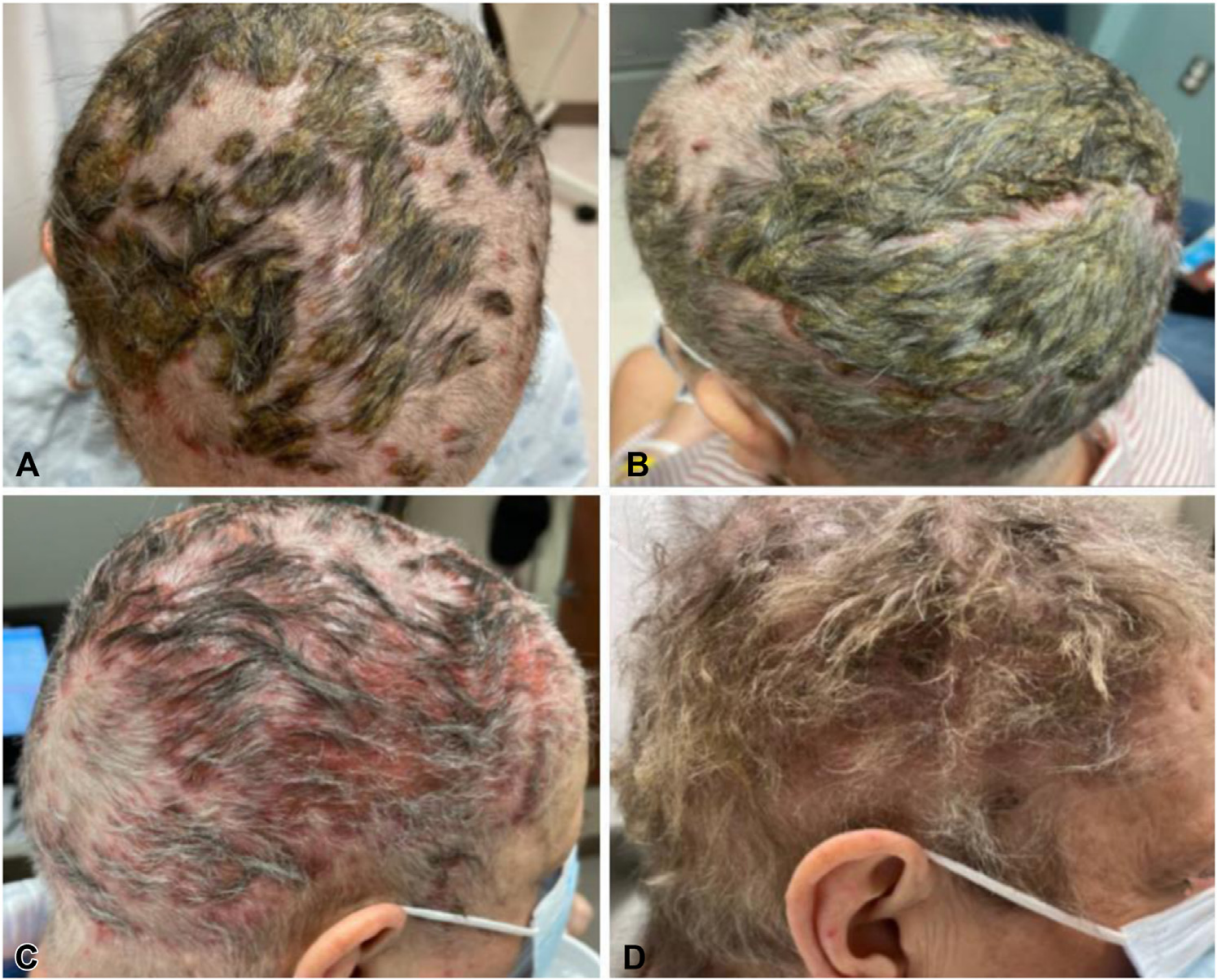
Case 2. **A,** Erosive pustular dermatosis-like eruption at month 5 of amivantamab therapy with crusted erosions and nonscarring alopecia. **B,** Worsening symptoms at month 7 with thick, yellow-brown crusts of erosions with alopecia. **C,** Improving symptoms with regrowth of hair 6 weeks after cessation of amivantamab. **D,** Healed eruption with regrowth of hair 2.5 months after discontinuation of amivantamab.

A 73-year-old woman with low-grade small cell lung carcinoma presented to the clinic with an acneiform eruption on the scalp and trunk 2 months after initiation of amivantamab. She also had no past history of radiation or actinic damage to the scalp. Her symptoms improved with oral prednisone and trimethoprim-sulfamethoxazole. However, 4 months into amivantamab therapy, she now presented with tender, matted, yellow, crusted plaques overlying hemorrhagic erosions on the scalp (Fig 4).

**Fig 5:**
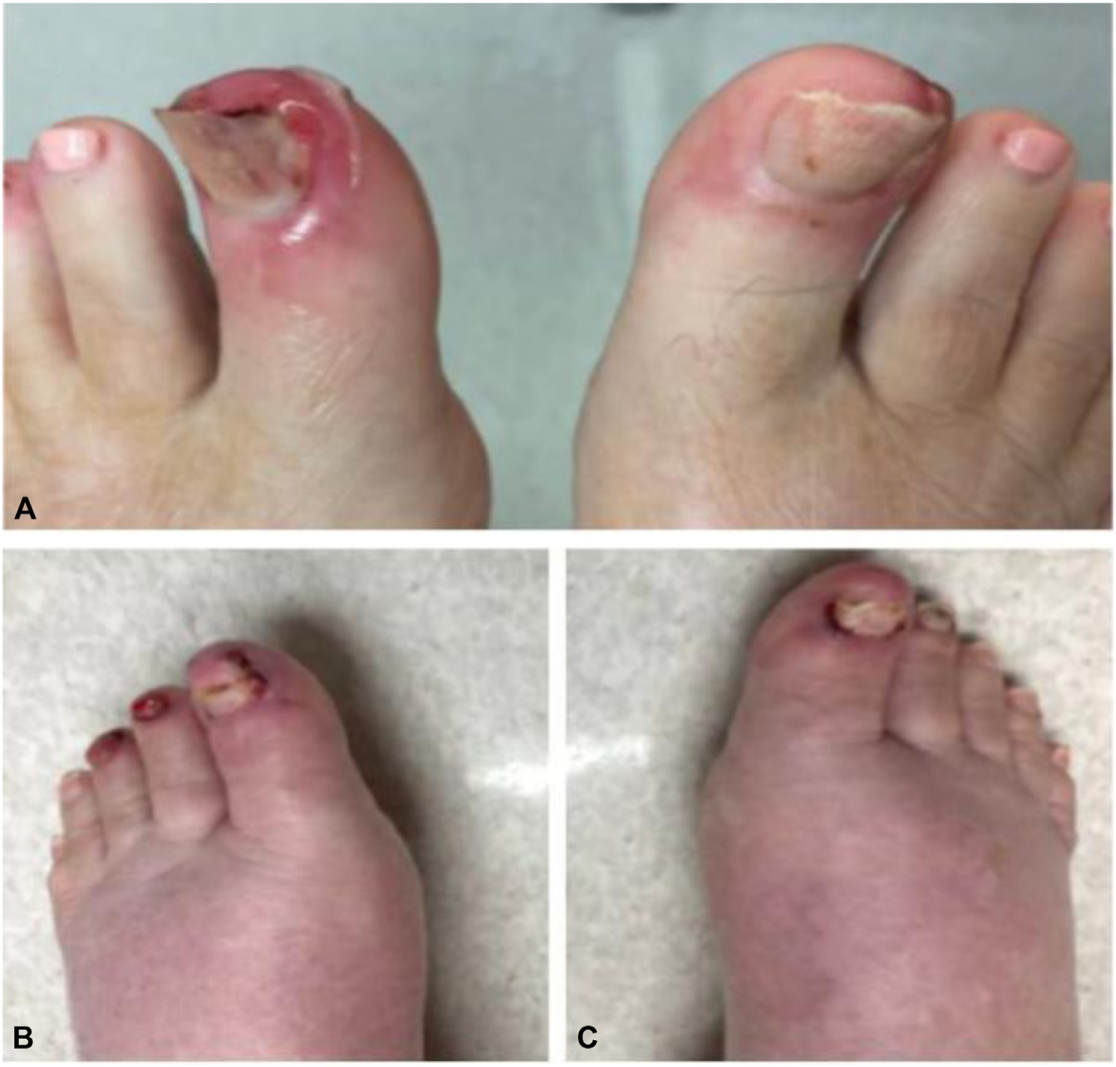
Case 2. **A,** Initial presentation of paronychia of several toes and pyogenic granulomas on bilateral first toes at month 6 of therapy with amivantamab. **B, C,** Continued paronychia and resolution of pyogenic granulomas 1 month after discontinuation of amivantamab.

Culture of the scalp was positive for *Staphylococcus aureus*. She also had paronychia of several toes, and pyogenic granulomas on bilateral first toes which were treated with cryotherapy (Fig 5).

The patient was started on prednisone, doxycycline, triamcinolone ointment, and clobetasol shampoo with 70% improvement in scalp lesions after 2 weeks but no improvement thereafter despite acitretin and isotretinoin. At 8 months of therapy with amivantamab, purulent draining lesions developed in her inframammary region. Amivantamab was discontinued because of progressive disease and persistent severe cutaneous toxicity. Her scalp lesions worsened with foul-smelling discharge over the following weeks. Improvement in the scalp and inframammary lesions was noted 6 weeks after cessation of amivantamab therapy.

## Case 3

**Fig 6:**
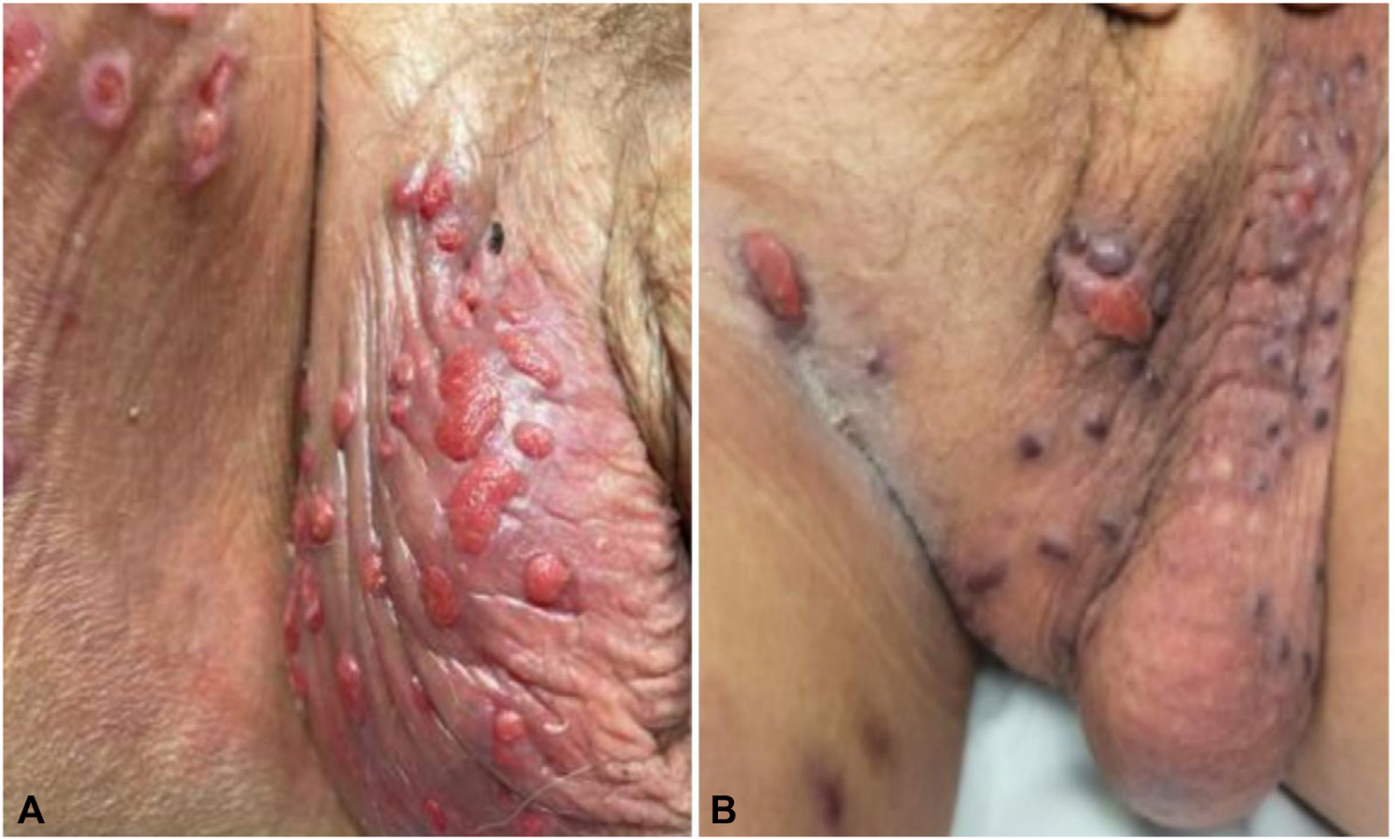
Case 3. **A,** Initial presentation of ulcerated, granulation lesions on the scrotum at month 9 of amivantamab therapy. **B,** Improved scrotal lesions after 1 month of drug holiday.

A 66-year-old man with metastatic lung adenocarcinoma presented with ulcerated, granulation lesions on the scrotum and diffuse paronychia 9 months after initiation of amivantamab (Fig 6).

**Fig 7:**
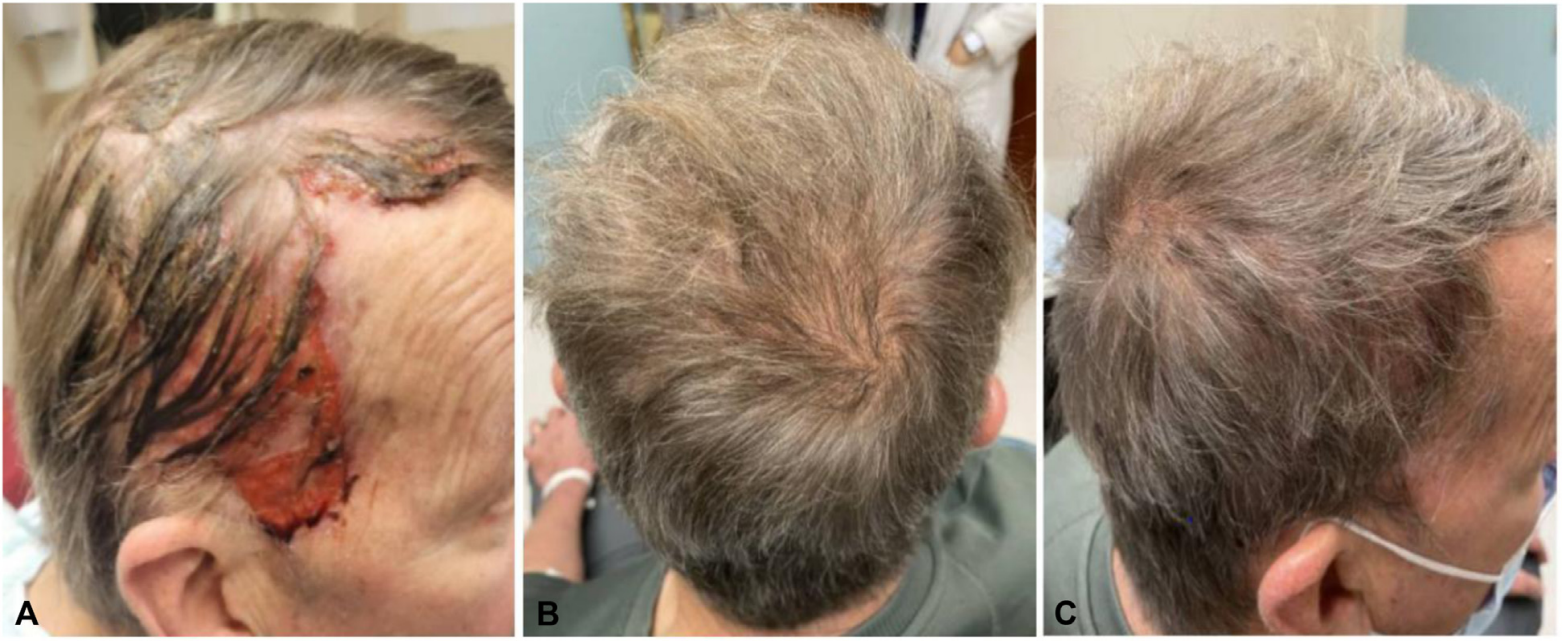
Case 3. Erosive pustular dermatosis-like eruption. **A,** On month 16 of amivantamab therapy with crusted hemorrhagic erosions on the frontal scalp with areas of alopecia. **B, C,** Healed eruption with regrowth of hair on month 20 of amivantamab.

Biopsy of the scrotal lesions revealed ulceration and florid granulation tissue. His scrotal ulcers were treated with barrier creams and topical desonide, and the paronychia was treated with silvadene and vinegar soaks. Over 1 year after starting amivantamab, the patient began to experience severe breakdown and maceration of the scalp. Examination revealed dense scale on the frontal and parietal scalp with erythematous plaques and areas of alopecia within the purulent, draining erosions (Fig 7).

He reported limited sun exposure and no history of blistering sunburns on the scalp. He was started on prednisone and acitretin, and amivantamab was ultimately held because of worsening scalp lesions and drainage. However, he was unable to tolerate the acitretin because of nausea. He was then started on oral dapsone, with improvement in his scalp, scrotal erosions, and paronychia. He was restarted on amivantamab with an increase in the interval between cycles from 2 weeks to 3 weeks. The scalp lesions continued to improve on dapsone with regrowth of his hair, and currently remain under good control (Fig 7). He continues on dapsone and fluocinolone, and his lung cancer remains stable.

## Discussion

Amivantamab is a bispecific monoclonal antibody targeting EGFR and mesenchymal-epithelial transition approved in 2021 for treatment of locally advanced or metastatic non–small cell lung cancer.^1^ Although the cytostatic mechanism of action of targeted therapy is believed to cause fewer adverse events compared with traditional chemotherapy, they are associated with a wide range of cutaneous toxicities that can be debilitating and cause interruption or cessation of anticancer treatment.^4^ EGFR inhibitors are widely implicated in cutaneous toxicities because of the integral role of EGFR in cellular signaling pathways in the skin. Initial reports from the phase I study evaluating amivantamab in patients with non–small cell lung cancer identified rashes and paronychia as 2 of the most common treatment-related adverse effects.^1^ In >50% of cases, an acneiform rash was reported mainly on the sebaceous areas in the scalp, face, and upper portion of the trunk. Nail changes, such as paronychia, are another highly reported adverse reaction, hypothesized to be due to EGFR inhibition on keratinocytes in the nail matrix.^5^

Our patients presented with acneiform rash and paronychia, the most common EGFR inhibitor–associated cutaneous reactions, in what appeared to be prodromal symptoms preceding the development of an EPD-like eruption on the scalp. The onset of this EPD-like eruption occurred between 4 and 13 months after initiation of amivantamab despite improvement in these prodromal symptoms. The insidious onset of this delayed progression to EPD-like eruption in our patients highlights the importance of close follow-up with these patients and early referral to dermatology with new or worsening cutaneous findings (Table I).

**Table I:** Summary of erosive pustular dermatosis-like eruption, other cutaneous adverse effects, and treatments in 3 patients treated with amivantamab

Variables	Case 1	Case 2	Case 3
Age/sex	62/F	73/F	66/M
Type of stage IV lung cancer	Adenocarcinoma	Small cell	Adenocarcinoma
Amivantamab course			
Dose	1050 mg	1050 mg	1050 mg
Other concurrent anticancer therapy	Osimertinib		
Drug holiday	X	X	X
Rechallenge	X		X∗
Permanent cessation	X	X	
Cancer progression on amivantamab	X	X	
EPD-like eruption			
Months to onset	4	4	13
*Staphylococcus aureus*–positive culture	X	X	
Improvement with drug holiday	X	N/A	X
Worsening with amivantamab rechallenge	X	N/A	
Resolution with amivantamab cessation	X	X	N/A
Control of EPD with amivantamab continuation	N/A	N/A	X†
Other cutaneous toxicities			
Acneiform rash	X	X	X
Paronychia	X	X	X
Pyogenic granuloma		X	
Other skin ulceration/erosion	X‡	X§	Xװ
Treatment modalities			
Topical antimicrobials			
Mupirocin	X		
Ketoconazole	X		
Systemic antimicrobials			
Doxycycline	X	X	X
Trimethoprim-sulfamethoxazole		X	
Dapsone			X
Steroids			
Fluocinolone	X		X
Triamcinolone		X	
Clobetasol		X	X
Prednisone	X	X	
Retinoids			
Isotretinoin	X	X	
Acitretin		X	

*EPD*, Erosive pustular dermatosis; *N/A*, not available.

∗ Restarted on amivantamab with the interval between cycles increased from 2 weeks to 3 weeks.

† Receiving 50 to 75 mg dapsone.

‡ Scalp nodule.

§ Inframammary lesions.

װ Scrotal lesions.

EPD is a rare chronic eruption typically associated with autoimmune disorders and predisposing factors such as photodamage and old age, possibly because of senescent immune dysregulation.^6^ It can occur anywhere on the skin and presents with sterile pustules, superficial erosions, and yellow-brown crusted lesions with associated scarring alopecia. Although the exact etiology remains unknown, EPD is typically associated with a history of local trauma because of actinic damage or epidermal atrophy. Immunotherapy and targeted therapies have been linked to the pathogenesis of EPD-like eruption of the scalp. EGFR inhibitors such as gefitinib, afatinib, and panitumumab have been linked to rare EPD-like reactions with scarring alopecia with reports presenting a few months to up to 3 years after initiation of therapy.^7^, ^8^, ^9^ Our review of the literature identified only 1 case of EPD of the scalp following treatment with an unspecified monoclonal antibody targeting both EGFR and mesenchymal-epithelial transition.^10^

The hair follicle is a site of immune privilege, hidden from the immune system, and disruption by actinic insult or mechanical trauma can lead to immune dysregulation and cicatricial hair loss.^2^ Treatment for EPD of the scalp is typically immunosuppressive with corticosteroids, steroid-sparing agents such as tacrolimus, and photodynamic therapy.^6^ Successful treatment has also been reported with vitamin A derivatives such as isotretinoin, known for its antiinflammatory effects and normalization of keratinization. In 2 of our patients, although various therapies were trialed, cessation of amivantamab was necessary for notable clinical improvement. In our third patient, continued improvement was seen with dapsone allowing the patient to remain on the same dose of amivantamab at a slightly increased interval between doses. The efficacy of dapsone in treating the EPD-like eruption is likely related to its antineutrophilic and antiinflammatory properties and should be considered early on at the first signs of scalp involvement.^11^

**Fig 8:**
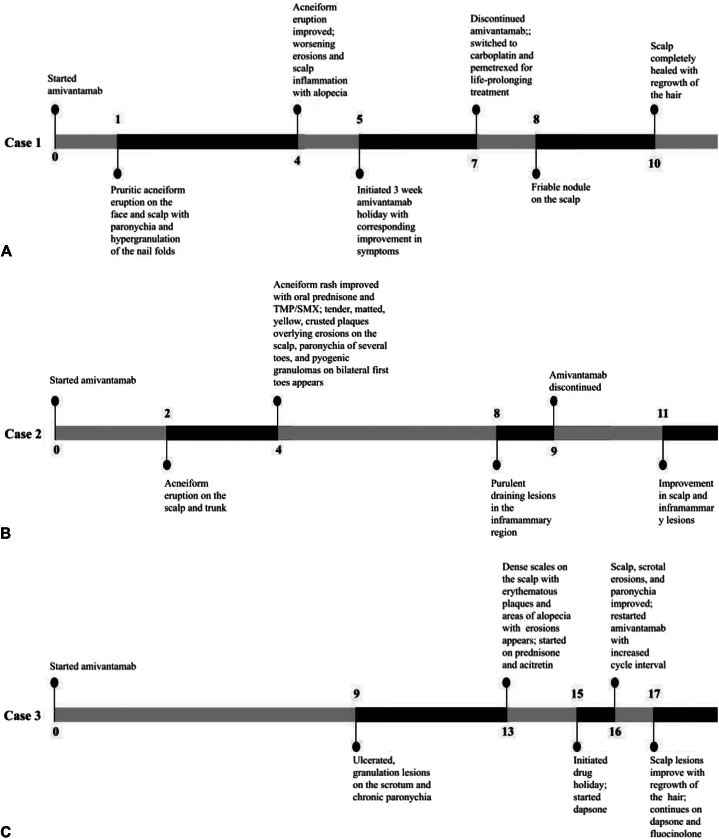
Timeline in months since initiation of amivantamab. **A,** Case 1. **B,** Case 2. **C,** Case 3.

EGFR inhibition likely leads to epithelial cell growth arrest with deranged, premature differentiation and resulting granulation tissue growth.^12^ In all 3 of our patients, rare granulation changes presented at various intervals during amivantamab therapy (Fig 8).

A large nodule was found on the scalp on month 8 of therapy in our first patient, pyogenic granulomas of bilateral halluces and inframammary erosions at 4 months and 8 months of therapy respectively in our second patient, and diffuse ulcerated scrotal lesions at 9 months of therapy in our third patient. Delay in diagnosis of EPD-like eruption because of its resemblance to other dermatologic disorders can lead to increased morbidity and significant psychosocial implications. Given the severity and abrupt nature of the eruption, it is important for dermatologists to have a high clinical suspicion of EPD-like eruptions with amivantamab use to facilitate prompt treatment.

## Conflicts of interest

None disclosed.
